# IDEAS (Integrate, Design, Assess, and Share): A Framework and Toolkit of Strategies for the Development of More Effective Digital Interventions to Change Health Behavior

**DOI:** 10.2196/jmir.5927

**Published:** 2016-12-16

**Authors:** Sarah Ann Mummah, Thomas N Robinson, Abby C King, Christopher D Gardner, Stephen Sutton

**Affiliations:** ^1^ Stanford Prevention Research Center Department of Medicine Stanford University School of Medicine Stanford, CA United States; ^2^ Behavioural Science Group Institute of Public Health University of Cambridge Cambridge United Kingdom; ^3^ Stanford Solutions Science Lab Department of Pediatrics Stanford University School of Medicine Stanford, CA United States; ^4^ Division of Epidemiology Department of Health Research & Policy Stanford, CA United States

**Keywords:** health behavior, design thinking, user-centered design, behavioral theory, behavior change techniques, digital interventions, mobile phones, digital health, telemedicine, diet, exercise, weight loss, smoking cessation, medication adherence, sleep, obesity

## Abstract

Developing effective digital interventions to change health behavior has been a challenging goal for academics and industry players alike. Guiding intervention design using the best combination of approaches available is necessary if effective technologies are to be developed. Behavioral theory, design thinking, user-centered design, rigorous evaluation, and dissemination each have widely acknowledged merits in their application to digital health interventions. This paper introduces IDEAS, a step-by-step process for integrating these approaches to guide the development and evaluation of more effective digital interventions. IDEAS is comprised of 10 phases (empathize, specify, ground, ideate, prototype, gather, build, pilot, evaluate, and share), grouped into 4 overarching stages: Integrate, Design, Assess, and Share (IDEAS). Each of these phases is described and a summary of theory-based behavioral strategies that may inform intervention design is provided. The IDEAS framework strives to provide sufficient detail without being overly prescriptive so that it may be useful and readily applied by both investigators and industry partners in the development of their own mHealth, eHealth, and other digital health behavior change interventions.

## Introduction

Digital technology has rapidly and dramatically shifted how humans interact with the world and presents an unprecedented opportunity to develop, test, and widely disseminate effective health behavior change interventions. The prospect of modifying lifestyle behaviors, such as diet, physical activity, smoking, and sleep, to improve health outcomes has increasingly driven efforts in both academia and industry. However, achieving meaningful and sustained improvements in health behaviors has eluded researchers and industry players alike, and numerous challenges remain. Within industry, most digital health interventions are yet to incorporate theory-based strategies known to drive changes in health behaviors or undergo systematic testing to demonstrate their effectiveness [1-3]. Moreover, interventions are often plagued by rapidly declining retention rates [4], with a quarter of downloaded health apps used only once and three-quarters discontinued after the tenth use [5]. Within academia, interventions are more often grounded in behavioral theory and tested for their efficacy [6]. However, they face similar challenges around declining retention rates, and the rapid pace of new technology development makes it increasingly difficult for researchers to develop, pilot-test, and evaluate interventions before such technologies become outdated or obsolete [7,8]. In addition, researcher-driven technologies often do not benefit from numerous rapid cycles of fine-tuning based on user feedback nor do they usually become widely disseminated among the broader public [9].

In light of these challenges, investigators have called for digital health interventions to be (1) grounded in behavioral theory [1-3], (2) grounded in an in-depth qualitative understanding of the target population [10], (3) rapidly and iteratively developed with multiple stages of user feedback [11,12], (4) subject to rigorous evaluation [8,13], and (5) widely disseminated [14]. However, published frameworks to guide the development of such technologies are disparate and no single approach integrates these elements. In addition, specific recommendations for integrating principles from behavioral theory are lacking in available frameworks. These gaps limit our ability to advance health behavior change research and practice. As our knowledge evolves in this young field, investigators have been called on to publish the methods they use to develop interventions to further advance the field [15]. This paper responds to that call and aims to build on prior models by combining behavioral theory, design thinking, and intervention evaluation and dissemination through a systematic framework to guide the development of more effective digital health interventions. It introduces Integrate, Design, Assess, and Share (IDEAS), an integrated 10-phase process.

### Essential Components of a Framework

#### Behavioral Theory

To maximize the potential efficacy of interventions to change health behavior, those who design interventions should have an understanding of theory or the hypothesized mechanisms underlying human behavior and behavior change [16]. Behavioral theory is widely acknowledged to be critical to the development of interventions to change health behavior [1-3,17-19] and increasing evidence suggests that health interventions grounded in theory are more effective than those without such theoretical foundations [19]. Among the most frequently used theories in health behavior research are social cognitive theory, the health belief model, and the transtheoretical model [20,21]. One approach to intervention development is to use one or more theories to identify the key constructs to be targeted in the intervention. For example, the health belief model suggests that a behavior change intervention should target perceived susceptibility, severity, benefits, and barriers. An alternative approach is to select behavior change techniques (eg, from those listed in the taxonomy of behavior change techniques [BCTs] [22]) and use these to construct the intervention. In principle, the two approaches could be used in combination, selecting techniques that are believed based on theory and/or evidence to be likely to produce change in the targeted behaviors.

#### Design Thinking

A recent consensus statement on the prevention of noncommunicable diseases emphasized the importance of using human-centered design, or *design thinking*, to develop effective and innovative interventions [23]. First coined by David Kelley, a founder of the design firm IDEO and the Stanford University Hasso Plattner Institute of Design (d.school), design thinking is intended to guide the development of more creative and innovative solutions [24]. The process has been summarized by the Stanford d.school as encompassing 5 phases: “empathize” (understand target population), “define” (identify goals and scope), “ideate” (brainstorm potential solutions), “prototype” (mock up primitive potential solutions), and “test” (gather feedback from target users) [25]. Design thinking starts by reframing the context for behavioral change around “what matters most” to a target group rather than “what’s the matter” with them [23]. This approach enables the design of more empathetic solutions that are more desirable to target populations. The importance of grounding behavioral health interventions in a deep understanding of a target population has been emphasized by experts in public health and health psychology [10,23]. Design thinking next involves rapidly and iteratively brainstorming, prototyping, and gathering user feedback on potential solutions. This process is similar to *user-centered design*, first presented by Norman and Draper [26], and involves gathering user feedback throughout intervention development, refining designs through prototyping and iteration, and including multidisciplinary skills and perspectives [27]. Core to design thinking is the notion that everyone has the potential to be highly creative and can learn to apply design-based approaches such as need-finding, brainstorming, prototyping, and iteration to unlock their creative potential [28]. It has been suggested that design thinking may increase the self-efficacy of those who practice the process [29] and frees even the most novice designers to generate more creative ideas and solutions. As a result, the process has been increasingly adopted among a diverse range of academics and industry players [24] and has been adapted to better serve the varied contexts in which it is used, particularly through discipline-specific frameworks and processes [30,31].

#### Evaluation and Dissemination

There have long been calls for the evaluation and dissemination of digital health interventions [8]. Rigorous evaluation is essential to judge whether or not an intervention achieves its desired effect. Published evaluations also contribute to the literature and evidence base, helping uncover the potential and challenges of using digital interventions to improve health outcomes [8,13]. Rigorous evaluation methods also enable more reliable conclusions. It has been recommended that the purpose of evaluation, the balance of potential benefits and risks, and the level of resource investment required by providers and users can help drive the necessary level of evidence needed [8]. As the UK Medical Research Council emphasizes, evaluation efforts should employ randomization whenever possible because it is considered the most robust approach to preventing several forms of bias. Without randomization to the intervention or a control or comparison condition, it is not possible to determine whether the intervention itself was responsible for the observed effects or whether a selected group of participants, for instance, happened to be highly motivated and might have improved without the intervention [19].

Moreover, dissemination is crucial if digital health interventions are to fulfill their potential. One of the great promises of digital interventions is their ability to reach broad segments of the population with minimal cost [14]. However, most publicly available apps to promote dietary behavior change have not been assessed in rigorous randomized controlled efficacy trials [32,33]. In turn, most digital health interventions that have demonstrated efficacy in peer-reviewed trials are not available to the public; instead, they have been created ad hoc for research purposes [32]. Thus, interventions that are publicly available have not been evaluated and those that have been evaluated are not publicly available [32]. As a result, there is a need for effective interventions to be more widely disseminated to populations that may benefit.

### Existing Frameworks and Limitations

Using the best combination of recommended approaches to guide intervention design is important if effective technologies to change health behavior are to be developed. Researchers often rely on published frameworks to guide them through the process of designing digital interventions [34]. However, currently available frameworks are numerous, disparate, and do not fully integrate behavioral theory, design thinking, and evaluation and dissemination. Although many electronic health (eHealth) frameworks exist, most envision their objective as guiding the development of technologies to facilitate medical or patient care (eg, patient-physician communication, access to medical records) rather than to modify health behavior [35,36]. For instance, Van Velsen et al [36] propose a “requirements development” approach in which stakeholder interviews are meant to lead directly to a list of technical specifications to be developed (eg, one-stop portal for patient information). This approach may be appropriate for building products to facilitate logistics or care provision in medical settings, but designing for behavior change is a different type of endeavor that requires thoughtful integration of behavioral theory and evidence.

Relatively few frameworks focus on guiding the development of digital interventions for the express purpose of changing health behavior. Among the frameworks that do are Yardley et al’s [10] person-based approach, Ludden et al’s [37] design research perspective, and Brown et al’s [38] health information technology usability evaluation model. Although each of these approaches provides valuable guidance for investigators, each focuses on a particular aspect of intervention development and none provides guidance on behavioral strategies that may be used in intervention design. Hekler et al’s [12] process, referred to as behavioral science-informed user experience design, combines a user-centered design approach with the use of behavioral theory-driven strategies. This process notably suggests the integration of user-centered and theory-based approaches, but it does not provide step-by-step guidance on how others may replicate the approach. Whittaker and colleagues [15] have proposed perhaps the most comprehensive step-by-step framework to date which involves 5 phases (focus groups, pretesting, pilot, randomized controlled trial [RCT], interviews) to guide overall mobile health intervention development and evaluation. Whittaker’s framework has been applied to the iterative development of numerous mobile health interventions [39-42], includes stages for user feedback and evaluation, and states the importance of using behavioral theory. However, it does not make use of design thinking approaches such as ideation, brainstorming, or rapid prototyping, nor does it include specific guidance on behavioral strategies that may inform intervention design. Although it has been suggested that design thinking and behavioral science can together inform the development of more effective digital health interventions [12], no published frameworks appear to combine behavioral theory, design thinking, and evaluation and dissemination into a comprehensive step-by-step process for guiding digital interventions to change health behavior. This gap limits our ability to advance health behavior change research and practice.

## IDEAS Framework

### Overview

**Figure 1:**
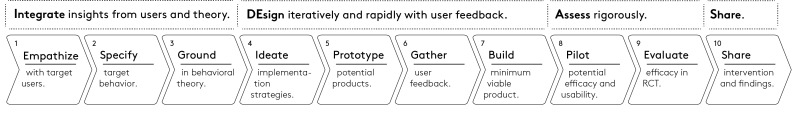
IDEAS (Integrate, Design, Assess, and Share) framework for developing digital health behavior change interventions.

To address the need for a framework that more fully integrates strengths from behavioral theory, design thinking, and evaluation and dissemination, we introduce IDEAS, a framework to better guide the development of digital health interventions to change behavior. IDEAS was informed by a multisector team of researchers, designers, and engineers, and was then applied to and refined in the iterative development of Vegethon, a mobile health (mHealth) intervention that demonstrated user acceptability and initial efficacy [43]. IDEAS consists of 10 phases: (1) empathize with target users, (2) specify target behavior, (3) ground in behavioral theory, (4) ideate implementation strategies, (5) prototype potential products, (6) gather user feedback, (7) build a minimum viable product, (8) pilot test to assess potential efficacy and usability, (9) evaluate efficacy in an RCT, and (10) share intervention and findings. These phases are grouped into 4 overarching categories: Integrate, Design, Assess, and Share (Figure 1).

**Figure 2:**
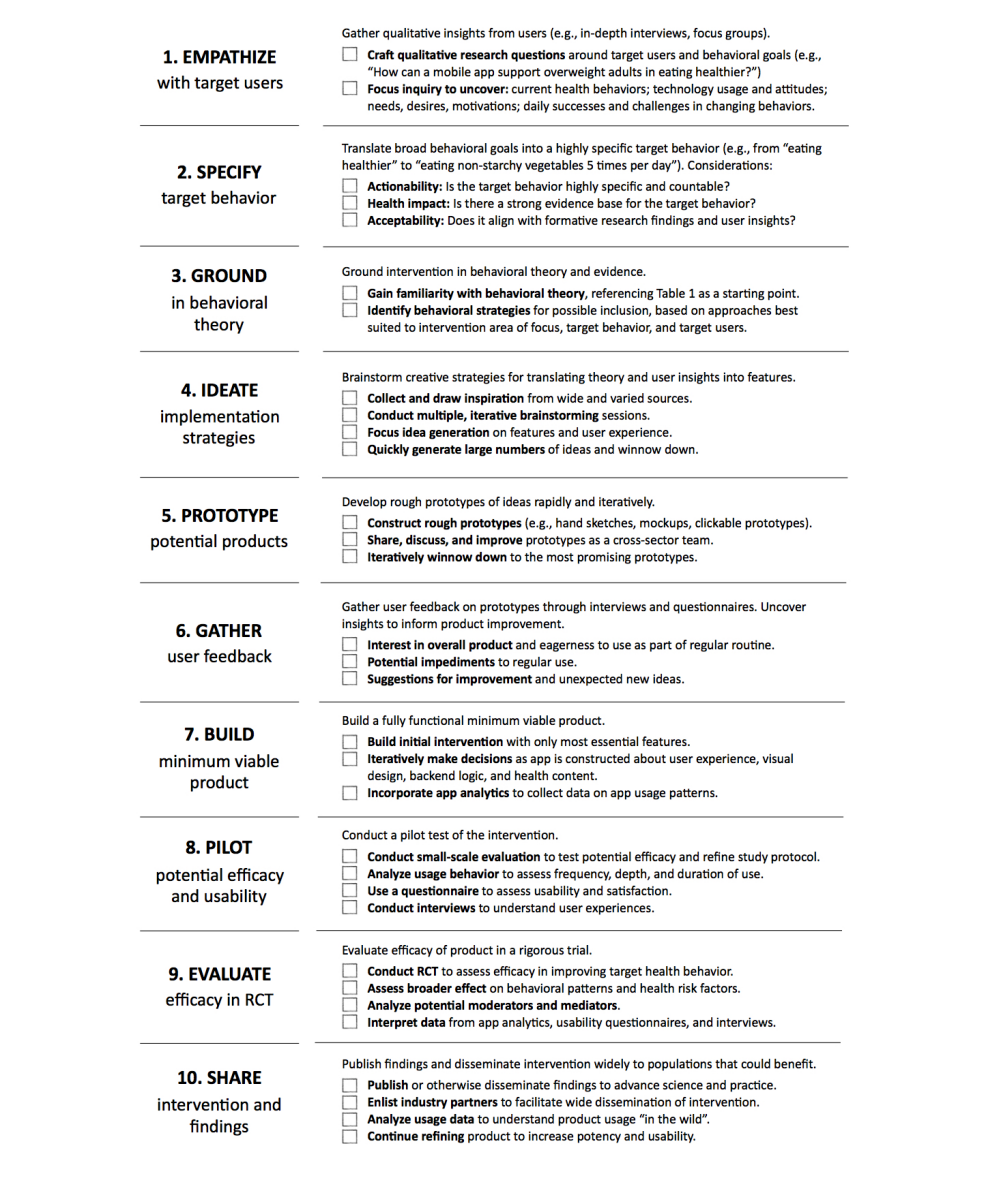
IDEAS (Integrate, Design, Assess, and Share) phases for developing digital health behavior change interventions.

IDEAS aims to facilitate the creation of more effective interventions by using strengths from a combination of disciplines at the intersection of digital health. The framework builds on design thinking and user-centered design in that it is iterative, engages multidisciplinary perspectives through a cross-sector team, includes stages for ideation and prototyping (phases 4, 5, 7), and integrates user insights throughout (phases 1 and 6). It focuses the design process around defining behavioral goals grounded in evidence (phase 2) and encourages the inclusion of theory-driven behavioral strategies both initially (phase 3) and throughout the design process. IDEAS integrates evaluation methods captured by the UK Medical Research Council framework [19] by emphasizing the importance of rigorously evaluating behavioral outcomes through both pilot and more substantially powered RCTs (phases 8 and 9). Finally, given the abundance of low-quality interventions currently available to the public, IDEAS concludes with a stage for dissemination (phase 10), which capitalizes on the readily scalable nature of digital interventions to provide access to target populations that may benefit. Dissemination also includes sharing findings with other audiences that can use the findings to advance the field and science of behavior change.

The 10 IDEAS phases are summarized in a step-by-step fashion in Figure 2. As with design thinking, although the IDEAS phases are presented sequentially, they are not necessarily intended to be carried out linearly, and projects are encouraged to loop back through the phases in an iterative fashion as different ideas and directions are explored and refined [44].

### Phase 1: Empathize With Target Users

The first 3 IDEAS phases (empathize, specify, and ground) aim to integrate insights from users and behavioral theory and orient the intervention development process around a measurable target behavior. These phases are primarily about information gathering to help inform the development in later stages of a more well-accepted, theory-driven intervention.

In phase 1, qualitative research is undertaken to gain a deeper understanding of the selected target population and their needs [10,45]. This need-finding stage can include observations, interviews, focus groups, and questionnaires [46]. Designers gain insight into users’ unmet or latent needs, which they may not necessarily be aware of or be able to articulate [47]. The goal of this stage is to move beyond identifying incremental improvements that users might be able to articulate (eg, refinements to a health app they already enjoy using) and to instead uncover deeper needs, values, and motivations that may help inspire more innovative and creative solutions [44]. For instance, nuanced insights, such as not wanting to feel like a failure or lacking family support for healthier cooking, while not directly suggestive of potential intervention solutions, may equip team members with the background and context necessary to develop an intervention that helps users feel supported with a virtual social support group and positive language. By engaging all members of the intervention development team in this qualitative research stage, it may be possible to guide the development of solutions that are more relevant and acceptable to the target population [48].

### Phase 2: Specify Target Behavior

Insights gathered from users help inform the next phase, in which a highly specific target behavior is selected. This target behavior defines both the purpose of the intervention as well as the outcomes by which the intervention will be judged. For instance, an initially broad intervention goal of “increase physical activity” may be refined to “take 10,000 steps each day.” The narrowing of the intervention goal in this way helps to focus the scope of idea generation and has been associated with success in the context of highly innovative concepts [49].

Multiple, and sometimes competing, factors may inform the selection of the target behavior. Insights from users in the previous phase may help investigators identify appropriate potential target behaviors that would be well accepted by individuals. A review of the literature may be conducted to identify the health benefits of possible target behaviors under consideration. For example, before deciding whether to target greater vegetable consumption or greater breakfast consumption, it would be advantageous to understand whether one behavior is more likely to have a greater health impact than the other. Some behaviors may be more susceptible to change than others, particularly if they are readily countable and therefore easier for users to self-monitor and modify. Some behaviors may also have the potential to produce a beneficial cascade effect, improving other health behaviors or aspects of the same health behavior [50]. Qualitative investigations have also shown that users may be more interested in technologies that frame behaviors as actions to increase as opposed to decrease (eg, increasing the number of days per week free of sugar-sweetened beverages vs cutting back on sugar-sweetened beverages) [51]. Thus, principles that may guide the selection of the intervention target behavior include (1) a behavior that is well accepted by the target population, (2) an evidence base demonstrating a significant health benefit to changing the target behavior, (3) a behavior that is highly specific and countable and therefore more actionable, (4) a behavior that is framed as something to increase versus decrease, and (5) optimally a behavior that if changed has the potential for producing a beneficial cascade effect on related and reinforcing behaviors.

### Phase 3: Ground in Behavioral Theory

Once a target behavior is identified, strategies are explored to ground intervention development in behavioral theory. The intervention design team may seek to gain familiarity with a range of theory-driven behavioral strategies available for inclusion. Strategies best suited to the target behavior and intervention delivery medium are identified for possible inclusion. Researchers may draw from behavioral theories, such as social cognitive theory [52], which are frequently used in academia to guide mobile health interventions [2]. Researchers may also draw from collections of theory-driven behavioral strategies, which have increasingly been introduced by investigators. For example, Michie et al’s [22] taxonomy summarizes 93 behavior change techniques such as goal setting, self-monitoring, feedback, prompts/cues, and action planning.

In our cumulative experience, we have also found it useful to apply a theory-driven process motivation lens during intervention development [53]. Process motivators, first introduced by Robinson [53], may be used to make the process itself of behavior change more engaging and intrinsically rewarding. Process motivators stand in contrast to *outcome motivators*, which focus on the eventual outcomes of behavior change such as weight loss, physical appearance, becoming more fit, and reducing risks of future chronic diseases [53]. Although outcome motivators have historically dominated medical and public health interventions (eg, smoking cessation interventions emphasizing reduced risk of lung cancer), such rational appeals are limited in their motivational power to initiate and sustain behavior changes [54,55]. Outcome motivators rely on results that are often delayed and difficult to achieve and maintain, reducing their motivational impact and an individual’s self-efficacy for behavior change [52,53,56]. By contrast, interventions that emphasize motivation for participating in the intervention itself, or the process of behavior change, may be more effective in initiating and sustaining behavior changes [53]. It has been suggested that process motivators, such as fun, taste, pride, choice, and challenge, can make the process of eating healthier or of engaging in physical activity more inherently enjoyable and desirable [53]. Table 1 presents examples of behavioral strategies using process motivation, adapted and extended from Robinson [53], and based on research on intrinsic motivation and interventions to change behavior [53,57-62], which may be useful to investigators in the development of their own interventions.

**Table 1:** Toolkit of behavioral strategies using process motivation to guide intervention design.

Behavioral strategy	Description
Challenge	Maintain optimal levels of moderate challenge (ie, not too hard, not too easy)
Choice/control	Provide objective and perceived choice and control over one’s environment and actions
Community	Provide social meaning (public recognition, identification with desirable group) for accomplishments
Competence	Provide immediate, frequent, clear, constructive, encouraging positive feedback following success
Competition	Facilitate social comparison and competition among individuals, groups, or teams
Context	Embed intervention into real/imaginary contexts with stories/characters
Curiosity	Provide sensory (color, taste, sound) and cognitive (mystery) curiosity and surprise
Growth mindset	Cultivate belief that behaviors/preferences (eg, for foods, activity levels) are malleable with effort
Identity	Facilitate an identity shift related to the behavior change (eg, someone who is now a runner)
Personalization	Personalize intervention using an individual’s name and personally relevant content
Pride	Cultivate pride and a sense of accomplishment
Piggybacking	Engage individuals in social movements (eg, animal rights) to harness deeper values
Reframing	Cast the purpose of a behavior in a more positive light to improve thoughts or feelings about it
Taste	Emphasize the taste and texture of healthier foods
Teamwork	Facilitate cooperation and teamwork among individuals, groups, or teams

Regardless of which behavioral theories or strategies are used or preferred by investigators, the goal of this phase is to consider ways in which behavioral theory can be incorporated into intervention development. Equipped with behavioral theory and strategies, subsequent stages enable the creative translation of such strategies into tangible and specific intervention components. Theory-driven insights can also be instrumental in helping guide the interpretation of user interview findings and addressing gaps in user understanding and awareness. At this stage, the team may wish to explore, discuss, compare, and reconcile insights from users and behavioral strategies in order to prepare for the brainstorming phase.

### Phase 4: Ideate Creative Implementation Strategies

The next 4 phases (ideate, prototype, gather, and build) involve a highly iterative design process that focuses idea generation around the target behavior and takes insights from users and behavioral theory into account throughout.

To begin, a series of group brainstorming sessions is held to ideate potential intervention solutions, components, and features. The agreed-upon target behavior focuses the scope of idea generation [25] and the aim is to generate a large number of diverse ideas that could influence the target behavior. Previously gathered user insights and behavioral theories help inspire the range of ideas. For instance, a “self-monitoring” behavioral strategy in the context of a target behavior to “increase daily steps” might inspire an idea to engage users in taking a photograph of the outdoor scenery each time they go for a run. User insights suggesting that individuals prefer outdoor running to other types of activities might lend further credibility to such an idea. Hundreds of ideas may be generated “ranging from the absurd to the obvious” (p 31 [44]). Adhesive notes can be used to capture different ideas (one idea per note), which can be displayed on a wall or board, and visual representations of ideas are encouraged to facilitate communication of ideas to other team members [44]. Divergent thinking is achieved through interdisciplinary teams engaging in structured brainstorming sessions. Team members are encouraged to generate ideas that build on prior ideas and/or are divergent from those already suggested. Deferring judgment is a central rule during this phase to encourage rather than discourage idea generation [63]. Throughout the process, weaker ideas drop off early on, whereas stronger ideas “naturally rise to the top” [44]. This phase is ideally undertaken with a cross-sector team to enable the generation of greater numbers of more varied and creative ideas. As brainstorming progresses, discussing and debating ideas during this highly creative stage is recommended because it has been associated with more novel innovations and produces a more thorough exploration of possible solutions [64].

### Phase 5: Prototype Potential Products

Prototypes are then created to share and discuss ideas with team members and to facilitate both further ideation (phase 4) and the rapid gathering of user feedback (phase 6). The prototyping phase is exemplified by the adage “enlightened trial and error outperforms the planning of flawless intellect” (p 1 [65]). Prototypes may be sketched, hand crafted using primitive materials, and/or developed into clickable mockups of digital interfaces. In the earliest stages, low-cost, low-fidelity (ie, “quick and dirty”) prototypes are developed rapidly to gather feedback from users early and often. This approach allows intervention designers to quickly and cheaply gather feedback on many different possible intervention approaches before investing significant resources in any one particular approach or suboptimal solution [25,66]. Experimental studies have demonstrated that the act of developing multiple prototypes in parallel (vs sequentially) leads to objectively stronger results [65]. In one experiment, when novice designers were instructed to develop multiple prototypes (vs a single prototype) before obtaining user feedback, designs overall tended to be more divergent and final prototypes were superior as measured by click-through data and blinded expert ratings. These findings were explained by qualitative data suggesting that parallel prototyping reduced fixation on a particular idea and encouraged designers to instead explore multiple directions before optimizing in any one direction. By contrast, sequential prototyping implicitly encouraged the refinement of the initial prototype at the expense of exploration of more divergent alternatives [29]. Although time constraints often lead teams to focus on the realization of a single idea rather than the iteration of multiple ideas [67], prototype iteration has been shown to lead to objectively stronger final products even when a team is under time constraints [65].

### Phase 6: Gather User Feedback

User feedback is then gathered on prototypes. Methods may include informal conversations, usability tests, surveys, and in-depth qualitative interviews. For instance, during a usability test of an early prototype, a researcher may observe participants use the intervention and participants may be asked to think aloud by providing commentary during the process. These methods of inquiry seek to uncover users’ interests in the overall product and eagerness to use it as part of their typical routine, potential impediments to usage on a regular basis, suggestions for improvement, and unexpected new ideas or opportunities. When this phase is initially employed in the design process, the focus is on quickly gathering initial user impressions and inspiring further divergent ideation. As this phase is repeated in quick succession with the ideate and prototype phases such that the phases inform one another [68], the goal is increasingly to inform concept refinement and focus. Thus, the ideate, prototype, and gather phases are carried out in a fluid and iterative fashion until a more refined design solution is reached [25].

### Phase 7: Build a Minimum Viable Product

Next, a fully functional minimum viable product (MVP) is developed to facilitate early learning from users. This stage focuses on a level of detail beyond the prototype stage with decisions made regarding user experience, visual design, content, and logic (eg, to calculate graphs, to trigger push notifications). Analytics are built into the product to enable the collection of a rich dataset capturing patterns of usage. The use of analytics such as metadata may be applied in the development of just-in-time, adaptive interventions (JITAIs), in which real-time data are used to tailor an intervention to the dynamically changing needs of a user [69,70]. The practical challenges and realities of building a digital intervention surface numerous decisions that need to be made with the technical developers. A multisector team can include perspectives to help ensure that the influence of behavioral theory, user needs and desires, and technical, financial, and practical feasibility are properly balanced and reconciled throughout the decision-making process. A core tenet of the MVP concept is to avoid wasting precious time and resources on perfecting a product that may be substantially altered in subsequent phases based on user feedback [71]. The goal, therefore, is to quickly develop only a minimum version of the product necessary to facilitate pilot testing among users, without incorporating additional unnecessary features. However, this industry-driven tenet must be balanced with the common assumption among behavioral scientists that many behavioral strategies are most effective when used in concert, rather than in isolation [39], which may encourage the inclusion of more behavioral strategies. Weighing these opposing priorities (ie, fewer features vs more theory-driven strategies) will determine how theory- and feature-rich the minimum viable intervention becomes at this stage. The degree to which an intervention is theory- and feature-rich will vary in each case and depend on the unique resource (eg, financial, human, time) constraints as well as the behavioral needs of the intervention. As with all phases in IDEAS, this phase can be conducted iteratively with other phases, particularly phase 6, to gather user feedback to help inform product decisions before pilot testing.

### Phase 8: Pilot Test

The final 3 phases (pilot, evaluate, and share) aim to assess and disseminate the final intervention and evaluation results. This process begins by subjecting the MVP to pilot testing to gather information on the usability, feasibility, and potential efficacy of the first viable version of the intervention [8,15,39]. For example, usability and satisfaction questionnaires may ask individuals on a 5-point scale how strongly they agree with statements such as “I have found this app easy to use,” “this product has motivated me to be more physically active,” or “I have had a hard time remembering to use this product” [72]. Analysis of product usage data helps identify usage patterns and peak usage times, which may inform notification timing. Qualitative and usability interviews lend insight into preferred features, problematic or confusing user flows, and opportunities for intervention modifications or improvements. Study design and implementation procedures, such as recruitment, enrollment, retention, and data collection, are tested and refined to avoid problems in subsequent larger-scale evaluation studies [73]. Randomization, which may [74] or may not [15] be used at this pilot stage, enables the potential efficacy of the intervention to be assessed [75]. Based on the wealth of information gathered during this stage, the intervention is further refined until it is ready for a larger-scale evaluation.

### Phase 9: Evaluate Efficacy

A sufficiently powered RCT is then conducted to test intervention efficacy. RCTs are considered the most robust study design for evaluating interventions due to their ability to minimize bias [8,19,76]. Outcomes may include measures of the original target health behavior; mediators, proximal behaviors, or conditions that may lie on the causal pathway to behavior change; and broader effects on related health behaviors and risk factors. Assessing possible moderators, or preexisting factors that may help define characteristics of users that respond more or less to the intervention, has also been recommended to better define the appropriate target audience for ultimate dissemination [77]. Given the rapidly evolving nature of mobile technology and the time involved in carrying out a rigorous evaluation study, the digital intervention may need to be continuously refined during the trial to ensure that it does not become obsolete by the time the trial is completed [13]. Researchers need to weigh the advantages of such an approach with the potential limitations to internal validity that are introduced if the intervention is modified too dramatically. To yield reliable and valid results, several best practices for conducting an RCT are followed. Randomization is used to eliminate conscious and unconscious selection bias in the assignment of participants to the intervention versus a control condition [78]. Proper randomization is achieved when the allocation sequence is unpredictable and concealed, such that the researcher enrolling participants does not know in advance which treatment the next participant will receive [79,80]. The widely recommended “intention-to-treat” analysis approach is used to preserve the benefits of randomization, in which all randomized participants are included in the analysis and retained in the original groups to which they were allocated, regardless of adherence to the digital intervention, missing data, or dropouts [81,82]. Data collectors and researchers who perform statistical analyses are blinded to intervention assignment to prevent the introduction of biases in the interpretation or analysis of data. For further guidance on RCTs, researchers may consult the evidence-based Consolidated Standards of Reporting Trials (CONSORT) 2010 guidelines [83].

### Phase 10: Share Widely

There are two types of sharing implied in the IDEAS framework. First, learnings and evaluation results should be disseminated to both researchers and digital health intervention developers to help advance the field and improve the effectiveness of future interventions. For example, the evaluation trial’s findings should be published, including a description of the final intervention and its theoretical basis and/or use of behavior change techniques, such as those using standardized language and descriptors from a taxonomy of behavior change techniques [22]. The second type of sharing involves disseminating efficacious interventions more broadly with appropriate target audiences. One of the greatest promises of digital health interventions is their potential to be widely scaled to users at a relatively low cost [84]. Thus, once intervention efficacy has been demonstrated, the intervention is disseminated to the broader population that may benefit the most, as demonstrated in the evaluation. Scaling an intervention for wide dissemination is not a trivial undertaking. Health-related intervention goals may be less salient in certain dissemination settings where, for instance, health care providers, health insurers, and/or employers may be more motivated by cost savings. The systems architecture (eg, servers, databases, storage systems) of an intervention may need to be adjusted to account for greater traffic at scale. Further intervention refinements may be warranted based on dissemination goals [10]. Cost considerations, including marketing, maintenance, and ongoing implementation, may significantly influence adoption [14]. Academic researchers may wish to form strategic partnerships with relevant public and private sector organizations to support the more effective and sustainable dissemination of the final intervention [84].

### How to Apply IDEAS

#### Engage a Multidisciplinary Team

Digital health is increasingly acknowledged to be an inherently transdisciplinary endeavor in which involving user-centered designers, programmers, behavioral researchers (particularly those working in the area of behavioral intervention development), and industry partners is crucial [9,11]. The effectiveness of digital health interventions rests on multiple diverse factors including esthetic design, behavioral theory, evidence grounding, user centeredness, technical capacity, and demonstrated efficacy. Only a true multidisciplinary team will have the knowledge and expertise to address these complex factors in tandem [27]. Engaging all team members throughout the full intervention development process may help the team proceed through each of the phases iteratively, nimbly, collaboratively, and with greater buy-in at all stages. This practice can also help ensure that all team members understand users’ needs and the theoretical grounding underpinning the intervention. Finally, enlisting the full multidisciplinary team may facilitate the generation of more highly divergent ideas and prototypes, which has been shown to lead to more creative and efficacious final intervention designs [64].

#### Use a Flexible, Iterative Approach

As with design thinking, the teams that stand to benefit the most from applying the IDEAS framework are those who will apply the stages flexibly, pursuing multiple stages in parallel, in combination, more than once, and/or iteratively, as new directions are explored and refined [68]. For example, some high-performing teams have been shown to combine, in particular, the ideate, prototype, and gather phases, rapidly shifting between them as needed. A single brainstorming session (ideate phase) may flow directly into the use of highly primitive prototypes (prototype phase) to communicate ideas to team members, generate new ideas, and/or gather user feedback (gather phase) to facilitate further brainstorming (ideate phase) [68]. In this way, the 10 IDEAS phases are more like “a system of overlapping spaces, rather than a sequence of orderly steps” as design thinking has been described [44].

#### Transition Between Divergence and Convergence

A key consideration in applying the framework involves balancing the opposing processes of divergent and convergent thinking. Idea divergence, or the generation of highly diverse and varied ideas, is critical during early stages of the intervention development process to fully consider the range of user experiences and possible solutions. However, idea convergence, or the narrowing of possible ideas, becomes equally important to identify a target behavior and refine potential solutions without being hampered by a continual revision of prior decisions which may impede progress. High-performing teams have been shown to shift their behavior nimbly and repeatedly as needed throughout the design process, exhibiting greater debate and divergence during “concept generation” stages and less debate and more focused attention during “concept selection” stages [68]. In a space where digital technology is young and the best solutions are likely yet to be uncovered, a highly divergent approach in brainstorming possible solutions may maximize the likelihood of identifying the most potent solutions. As the design process progresses, successful teams may find it advantageous to transition from divergence to convergence, particularly once a set of possible solutions have been identified for refinement [85].

## Discussion

### Overview

This paper introduces IDEAS, a framework to guide the design, development, and evaluation of digital interventions to change health behavior. It includes a summary of behavioral strategies that may be useful for intervention developers seeking to apply IDEAS in the development of their own interventions. IDEAS is among the few frameworks that aim to guide the development of digital interventions to change health behavior. It builds on Whittaker et al’s [15] framework by incorporating phases for ideation and prototyping that may contribute to more creative interventions and by providing guidance on behavioral strategies that may inform more effective technologies. It draws on strengths from Yardley et al’s [10] person-based approach through a focus on qualitative inquiry and Hekler et al’s [12] behavioral science-informed user experience design model through a focus on behavioral strategies and user-centered design. Although the importance of combining behavioral theory and design thinking has been emphasized [12], IDEAS appears to be the first framework to provide step-by-step guidance on integrating these approaches.

### Strengths

Among the strengths of IDEAS is its provision of a toolkit of behavioral strategies grounded in process motivation, which may aid and inspire intervention design. These strategies include theory-driven approaches not presently captured by the taxonomy of behavior change techniques [22] and may serve as a useful additional resource for intervention developers seeking to develop their own solutions. Moreover, theory by nature is abstract [21] and intervention developers may not know how to translate such insights into concrete intervention features. The IDEAS framework fills this gap by suggesting that ideating, prototyping, and gathering user feedback may be used iteratively and in quick succession to incrementally translate theories into highly relevant and practical intervention components. IDEAS also addresses the topic of creativity, which has been given considerably less attention in the mHealth and eHealth design literature [36]. Through structured brainstorming sessions and rapid prototyping with user feedback, intervention developers aiming to incorporate greater amounts of creativity into their designs are provided with practical guidance on how to do so. In addition, by integrating user insights throughout the intervention development process, IDEAS helps guard against an unexpected mismatch between intervention goals and user goals, which may lead to low user satisfaction and poor adherence to an intervention [37,86]. Finally, the promise of digital interventions lies in their potential for reaching broad segments of the population [14]; where other frameworks have neglected dissemination altogether, the inclusion of dissemination as a key phase may help more intervention developers take this stage into consideration to advance the field overall and produce greater population impacts on health.

### Limitations

Several limitations to this framework exist. Despite research exploring the application of design-based approaches among novice designers [68], less-experienced users may find it difficult to effectively apply some of the suggested methods. There are practical challenges inherent to working with a multidisciplinary team and team members may disagree in a counterproductive manner or find it difficult to advance potential intervention solutions. The behavioral strategies presented are not exhaustive. The framework does not suggest the randomized evaluation of isolated intervention components [87]. However, it is commonly argued that behavioral strategies work best in concert with one another; thus, evaluating individual intervention components separately may not necessarily be advantageous in this context [39]. This framework is not the only approach to intervention development and many other useful approaches may complement or advance IDEAS. Future research is recommended to systematically evaluate the IDEAS framework.

### Conclusions

The IDEAS framework is proposed to guide the design, development, and rigorous evaluation of more creative and effective digital health interventions. It integrates behavioral theory, design thinking and user-centered design, and evaluation and dissemination, and summarizes a list of theory-driven behavioral strategies that may be useful to intervention developers. IDEAS is intended to accelerate the translation of behavioral theory and evidence into industry practice where most digital health interventions are born. Other researchers who use IDEAS or alternative frameworks are encouraged to report on their processes and outcomes so that we and others may learn from their experiences and continue to improve the quality, efficacy, and effectiveness of our digital health interventions.

## Abbreviations

d.school: Stanford University Hasso Plattner Institute of Design
eHealth: electronic health
IDEAS: Integrate, Design, Assess, and Share
JITAI: just-in-time, adaptive intervention
mHealth: mobile health
MVP: minimum viable product
RCT: randomized controlled trial

## References

[1] Azar KM, Lesser LI, Laing BY, Stephens J, Aurora MS, Burke LE, Palaniappan LP (2013). Mobile applications for weight management: theory-based content analysis. Am J Prev Med.

[2] Riley WT, Rivera DE, Atienza AA, Nilsen W, Allison SM, Mermelstein R (2011). Health behavior models in the age of mobile interventions: are our theories up to the task?. Transl Behav Med.

[3] Pagoto S, Schneider K, Jojic M, DeBiasse M, Mann D (2013). Evidence-based strategies in weight-loss mobile apps. Am J Prev Med.

[4] Chen J, Cade JE, Allman-Farinelli M (2015). The most popular smartphone apps for weight loss: a quality assessment. JMIR Mhealth Uhealth.

[5] McLean V http://www.prweb.com/releases/2011/04/prweb5268884.htm.

[6] Payne HE, Lister C, West JH, Bernhardt JM (2015). Behavioral functionality of mobile apps in health interventions: a systematic review of the literature. JMIR Mhealth Uhealth.

[7] Baker TB, Gustafson DH, Shah D (2014). How can research keep up with eHealth? Ten strategies for increasing the timeliness and usefulness of eHealth research. J Med Internet Res.

[8] Robinson T, Patrick K, Eng T, Gustafson D (1998). An evidence-based approach to interactive health communication: a challenge to medicine in the information age. Science Panel on Interactive Communication and Health. JAMA.

[9] Pagoto S, Bennett GG (2013). How behavioral science can advance digital health. Transl Behav Med.

[10] Yardley L, Morrison L, Bradbury K, Muller I (2015). The person-based approach to intervention development: application to digital health-related behavior change interventions. J Med Internet Res.

[11] Tate EB, Spruijt-Metz D, O'Reilly G, Jordan-Marsh M, Gotsis M, Pentz MA, Dunton GF (2013). mHealth approaches to child obesity prevention: successes, unique challenges, and next directions. Transl Behav Med.

[12] Hekler E, King A, Banerjee B, Robinson T, Alonso M, Cirimele J (2011). A case study of BSUED: behavioral science-informed user experience design. Proceedings of the SIGCHI Conference Extended Abstracts on Human Factors in Computing Systems.

[13] Kumar S, Nilsen WJ, Abernethy A, Atienza A, Patrick K, Pavel M, Riley WT, Shar A, Spring B, Spruijt-Metz D, Hedeker D, Honavar V, Kravitz R, Lefebvre RC, Mohr DC, Murphy SA, Quinn C, Shusterman V, Swendeman D (2013). Mobile health technology evaluation: the mHealth evidence workshop. Am J Prev Med.

[14] Bennett GG, Glasgow RE (2009). The delivery of public health interventions via the Internet: actualizing their potential. Annu Rev Public Health.

[15] Whittaker R, Merry S, Dorey E, Maddison R (2012). A development and evaluation process for mHealth interventions: examples from New Zealand. J Health Commun.

[16] Davis R, Campbell R, Hildon Z, Hobbs L, Michie S (2015). Theories of behaviour and behaviour change across the social and behavioural sciences: a scoping review. Health Psychol Rev.

[17] Campbell M, Fitzpatrick R, Haines A, Kinmonth AL, Sandercock P, Spiegelhalter D, Tyrer P (2000). Framework for design and evaluation of complex interventions to improve health. BMJ.

[18] Campbell NC, Murray E, Darbyshire J, Emery J, Farmer A, Griffiths F, Guthrie B, Lester H, Wilson P, Kinmonth AL (2007). Designing and evaluating complex interventions to improve health care. BMJ.

[19] Craig P, Dieppe P, Macintyre S, Michie S, Nazareth I, Petticrew M, Medical Research Council Guidance (2008). Developing and evaluating complex interventions: the new Medical Research Council guidance. BMJ.

[20] Painter JE, Borba CP, Hynes M, Mays D, Glanz K (2008). The use of theory in health behavior research from 2000 to 2005: a systematic review. Ann Behav Med.

[21] Glanz K, Bishop DB (2010). The role of behavioral science theory in development and implementation of public health interventions. Annu Rev Public Health.

[22] Michie S, Richardson M, Johnston M, Abraham C, Francis J, Hardeman W, Eccles MP, Cane J, Wood CE (2013). The behavior change technique taxonomy (v1) of 93 hierarchically clustered techniques: building an international consensus for the reporting of behavior change interventions. Ann Behav Med.

[23] Matheson GO, Klügl M, Engebretsen L, Bendiksen F, Blair SN, Börjesson M, Budgett R, Derman W, Erdener U, Ioannidis JP, Khan KM, Martinez R, van Mechelen W, Mountjoy M, Sallis RE, Schwellnus M, Shultz R, Soligard T, Steffen K, Sundberg CJ, Weiler R, Ljungqvist A (2013). Prevention and management of noncommunicable disease: the IOC Consensus Statement, Lausanne 2013. Clin J Sport Med.

[24] Kelley T, Kelley D (2013). Creative Confidence: Unleashing the Creative Potential Within All of Us.

[25] (2011). Stanford d.school: Design Thinking Bootcamp Bootleg.

[26] Norman D, Draper S (1986). User-centred system design: new perspective on human-computer interaction.

[27] Hermawati S, Lawson G (2014). Managing obesity through mobile phone applications: a state-of-the-art review from a user-centred design perspective. Pers Ubiquit Comput.

[28] Martin R (2009). Design of Business: Why Design Thinking is the Next Competitive Advantage.

[29] Dow SP, Glassco A, Kass J, Schwarz M, Schwartz DL, Klemmer SR (2010). Parallel prototyping leads to better design results, more divergence, and increased self-efficacy. ACM Trans Comput-Hum Interact.

[30] Katoppo M, Sudradjat I (2015). Combining Participatory Action Research (PAR) and Design Thinking (DT) as an alternative research method in architecture. Procedia Soc Behav Sci.

[31] Quade S, Schlueter O (2014). Adapting design thinking for media prototyping: innovative collaboration at universities and workplaces. ICERI2014 Proceedings.

[32] Fiordelli M, Diviani N, Schulz PJ (2013). Mapping mHealth research: a decade of evolution. J Med Internet Res.

[33] Coughlin SS, Whitehead M, Sheats JQ, Mastromonico J, Hardy D, Smith SA (2015). Smartphone applications for promoting healthy diet and nutrition: a literature review. Jacobs J Food Nutr.

[34] Horvath KJ, Ecklund AM, Hunt SL, Nelson TF, Toomey TL (2015). Developing Internet-based health interventions: a guide for public health researchers and practitioners. J Med Internet Res.

[35] van Gemert-Pijnen JE, Nijland N, van LM, Ossebaard HC, Kelders SM, Eysenbach G, Seydel ER (2011). A holistic framework to improve the uptake and impact of eHealth technologies. J Med Internet Res.

[36] Van Velsen L, Wentzel J, Van Gemert-Pijnen JE (2013). Designing eHealth that matters via a multidisciplinary requirements development approach. JMIR Res Protoc.

[37] Ludden GD, van Rompay TJ, Kelders SM, van Gemert-Pijnen JE (2015). How to increase reach and adherence of web-based interventions: a design research viewpoint. J Med Internet Res.

[38] Brown W, Yen P, Rojas M, Schnall R (2013). Assessment of the Health IT Usability Evaluation Model (Health-ITUEM) for evaluating mobile health (mHealth) technology. J Biomed Inform.

[39] Fjeldsoe BS, Miller YD, O'Brien JL, Marshall AL (2012). Iterative development of MobileMums: a physical activity intervention for women with young children. Int J Behav Nutr Phys Act.

[40] Waterlander W, Whittaker R, McRobbie H, Dorey E, Ball K, Maddison R, Myers SK, Crawford D, Jiang Y, Gu Y, Michie J, Ni MC (2014). Development of an evidence-based mHealth weight management program using a formative research process. JMIR Mhealth Uhealth.

[41] Whittaker R, Dorey E, Bramley D, Bullen C, Denny S, Elley CR, Maddison R, McRobbie H, Parag V, Rodgers A, Salmon P (2011). A theory-based video messaging mobile phone intervention for smoking cessation: randomized controlled trial. J Med Internet Res.

[42] Whittaker R, Merry S, Stasiak K, McDowell H, Doherty I, Shepherd M, Dorey E, Parag V, Ameratunga S, Rodgers A (2012). MEMO--a mobile phone depression prevention intervention for adolescents: development process and postprogram findings on acceptability from a randomized controlled trial. J Med Internet Res.

[43] Mummah SA, Mathur M, King AC, Gardner CD, Sutton S (2016). Mobile technology for vegetable consumption: a randomized controlled pilot study in overweight adults. JMIR Mhealth Uhealth.

[44] Brown T, Wyatt J (2010). Design thinking for social innovation. Stanford Soc Innov Rev.

[45] Thondoo M, Strachan DL, Nakirunda M, Ndima S, Muiambo A, Källander K, Hill Z, In SG (2015). Potential roles of Mhealth for community health workers: formative research with end users in Uganda and Mozambique. JMIR Mhealth Uhealth.

[46] Hammersley M (1990). Reading Ethnographic Research: A Critical Guide.

[47] Peeters M, Megens C, Hummels C, Brombacher A (2013). Experiential probes: probing for emerging behavior patterns in everyday life.

[48] Gasparini A (2015). Perspective and use of empathy in design thinking.

[49] Lynna GS, Akgunb AE (2001). Project visioning: its components and impact on new product success. J Prod Innov Manag.

[50] Duhigg C (2012). The power of habit: why we do what we do in life and business.

[51] Dennison L, Morrison L, Conway G, Yardley L (2013). Opportunities and challenges for smartphone applications in supporting health behavior change: qualitative study. J Med Internet Res.

[52] Bandura A (1986). Social Foundations of Thought and Action.

[53] Robinson T, Dubé L, Bechara A, Dagher A, Drewnowski A, Lebel J, James P (2010). Stealth interventions for obesity prevention and control: motvating behavior change. Obesity Prevention: The Role of Brain and Society on Individual Behavior.

[54] Wansink B (2006). Mindless Eating: Why We Eat More Than We Think.

[55] Ariely D (2008). Predictably Irrational: The Hidden Forces that Shape our Decisions.

[56] Bandura A (1997). Self-Efficacy: The Exercise of Control.

[57] Lepper M, Master A, Yow W, Maehr ML, Karabenick SA, Urdan TC (2008). Intrinsic motivation in education. Advances in Motivation and Achievement, Volume 15: Social Psychological Perspectives.

[58] Robinson TN, Borzekowski DLG (2006). Effects of the SMART classroom curriculum to reduce child and family screen time. J Communication.

[59] Robinson TN (2010). Save the world, prevent obesity: piggybacking on existing social and ideological movements. Obesity (Silver Spring).

[60] Dweck C (2008). Mindset: The New Psychology of Success.

[61] Crum AJ, Langer EJ (2007). Mind-set matters: exercise and the placebo effect. Psychol Sci.

[62] Petrie K, Cameron L, Ellis C, Buick D, Weinman J (2002). Changing illness perceptions after myocardial infarction: an early intervention randomized controlled trial. Psychosom Med.

[63] Kelley T, Littman J (2005). The Ten Faces of Innovation: IDEO's Strategies for Defeating the Devil's Advocate and Driving Creativity Throughout Your Organization.

[64] Pelled LH, Eisenhardt KM, Xin KR (1999). Exploring the black box: an analysis of work group diversity, conflict, and performance. Admin Sci Quart.

[65] Dow S, Heddleston K, Klemmer S (2009). The efficacy of prototyping under time constraints. Proceedings of the Seventh ACM Conference on Creativity and Cognition.

[66] Vredenburg K, Isensee S, Righi C (2002). User-Centered Design: An Integrated Approach.

[67] Schrage M (1999). Serious Play: How the World's Best Companies Simulate to Innovate.

[68] Seidel V, Fixon S (2013). Adopting design thinking in novice mutlidisciplinary teams: the application and limits of design methods and reflexive practices. J Prod Innov Manage.

[69] Spruijt-Metz D, Nilsen W (2014). Dynamic models of behavior for just-in-time adaptive interventions. Lect Notes Comput Sc.

[70] Nahum-Shani S, Smith S, Tewari A, Witkiewitz K, Collins L, Spring B (2014). Just-in-Time Adaptive Interventions (JITAIs): An Organizing Framework for Ongoing Health Behavior Support (Technical Report No. 14-126).

[71] Ries E (2011). The Lean Startup: How Today's Entrepreneurs Use Continuous Innovation to Create Radically Successful Businesses.

[72] King AC, Hekler EB, Grieco LA, Winter SJ, Sheats JL, Buman MP, Banerjee B, Robinson TN, Cirimele J (2013). Harnessing different motivational frames via mobile phones to promote daily physical activity and reduce sedentary behavior in aging adults. PLoS One.

[73] Eldridge SM, Ashby D, Feder GS, Rudnicka AR, Ukoumunne OC (2004). Lessons for cluster randomized trials in the twenty-first century: a systematic review of trials in primary care. Clin Trials.

[74] Craig P, Dieppe P, Macintyre S, Michie S, Nazareth I, Petticrew M (2013). Developing and evaluating complex interventions: the new Medical Research Council guidance. Int J Nurs Stud.

[75] Nollen NL, Mayo MS, Carlson SE, Rapoff MA, Goggin KJ, Ellerbeck EF (2014). Mobile technology for obesity prevention: a randomized pilot study in racial- and ethnic-minority girls. Am J Prev Med.

[76] Evans D (2003). Hierarchy of evidence: a framework for ranking evidence evaluating healthcare interventions. J Clin Nurs.

[77] Kraemer HC, Frank E, Kupfer DJ (2006). Moderators of treatment outcomes: clinical, research, and policy importance. JAMA.

[78] Schulz KF (1998). Randomized controlled trials. Clin Obstet Gynecol.

[79] Schulz KF, Chalmers I, Hayes RJ, Altman DG (1995). Empirical evidence of bias. Dimensions of methodological quality associated with estimates of treatment effects in controlled trials. JAMA.

[80] Schulz KF, Chalmers I, Grimes DA, Altman DG (1994). Assessing the quality of randomization from reports of controlled trials published in obstetrics and gynecology journals. JAMA.

[81] Herman A, Botser IB, Tenenbaum S, Chechick A (2009). Intention-to-treat analysis and accounting for missing data in orthopaedic randomized clinical trials. J Bone Joint Surg Am.

[82] Hollis S, Campbell F (1999). What is meant by intention to treat analysis? Survey of published randomised controlled trials. BMJ.

[83] Moher D, Hopewell S, Schulz K, Montori V, Gøtzsche P, Devereaux P (2010). CONSORT 2010 Explanation and Elaboration: updated guidelines for reporting parallel group randomised trials. J Clin Epidemiol.

[84] Schneider F, Schulz DN, Pouwels LH, de Vries H, van Osch LA (2013). The use of a proactive dissemination strategy to optimize reach of an internet-delivered computer tailored lifestyle intervention. BMC Public Health.

[85] Gersick CJ (1989). Marking time: predictable transitions in task groups. Acad Manage J.

[86] Kelders SM, Van Gemert-Pijnen JE, Werkman A, Nijland N, Seydel ER (2011). Effectiveness of a Web-based intervention aimed at healthy dietary and physical activity behavior: a randomized controlled trial about users and usage. J Med Internet Res.

[87] Collins LM, Murphy SA, Strecher V (2007). The multiphase optimization strategy (MOST) and the sequential multiple assignment randomized trial (SMART): new methods for more potent eHealth interventions. Am J Prev Med.

